# Steller’s sea cow uncertain history illustrates importance of ecological context when interpreting demographic histories from genomes

**DOI:** 10.1038/s41467-022-31381-6

**Published:** 2022-06-28

**Authors:** Alberto A. Campos, Cameron D. Bullen, Edward J. Gregr, Iain McKechnie, Kai M. A. Chan

**Affiliations:** 1 Institute for Resources, Environment, and Sustainability, University of British Columbia, Vancouver, BC Canada; 2 SciTech Environmental Consulting, Vancouver, BC Canada; 3 Department of Anthropology, University of Victoria, Victoria, BC Canada; 4 Hakai Institute, Heriot Bay, Quadra Island, BC Canada; 5 Bamfield Marine Sciences Centre, Bamfield, BC Canada

**Keywords:** Biogeography, Palaeoecology

**arising from** F.S. Sharko et al. *Nature Communications* 10.1038/s41467-021-22567-5 (2021)

In their recent paper entitled *“*Steller’s sea cow genome suggests this species began going extinct before the arrival of Paleolithic humans*”*, Sharko et al.^1^ use novel genomic methods to infer the demographic history of this species. Based on a single specimen from the Commander Islands, the authors conclude that the species suffered a single catastrophic population decline approximately 400,000 years ago and was thus already on the verge of extinction well before human arrivals in the Late Pleistocene. Here we suggest their demographic assumptions warrant reinterpretation given the ecological barriers that likely structured sea cow populations along the North Pacific Rim. Our preliminary range simulations suggest that the Commander Is. population may have been physically isolated from others, making it unsuitable as a demographic inference for the entire sea cow North Pacific range. Under these assumptions, Sharko et al.’s findings are more likely indicative of the time since the isolation of this remnant population from the rest of the sea cow range, rather than representative of the population contraction of the species. This perspective highlights the importance of considering historical ecology and paleobiogeography when interpreting genomic data to infer past demographic histories.

Since Li and Durbin^2^ published their milestone paper on the development of pairwise sequentially Markovian coalescent (PSMC) models to infer species demographic histories from a single diploid genome, their approach has become extremely popular with iconic endangered (e.g., giant panda^3^) and extinct species (e.g., passenger pigeon^4^, woolly mammoth^5^). Although there are significant methodological questions, such as the effects that natural selection can have on vertebrate genomes^6^, and separating population size variation from population structure^7^, we believe this method will contribute in impactful ways to our understanding of species demographic histories, especially given the possibility of using few, or single specimens.

However, to appropriately substantiate assumptions, demographic analyses derived from such models must carefully consider long-term range fluctuations, as well as ecological and behavioural characteristics that would influence the species’ dispersal, distribution and consequently gene flow dynamics^8^. In this sense, although we were pleased to see these novel genetic methods applied to the demographics of marine megafauna in the North Pacific, particularly to Steller’s sea cow^1^, we believe an examination of this species’ ecology and biogeography lends clarity to the interpretation of their results.

The North Pacific-wide presence in the fossil record, the absence of visible physical barriers for dispersal and migration, and the sheer size of Steller’s sea cow (i.e., their whale-like appearance suggesting much greater mobility and navigational ability than they may have possessed^9,10^), have all contributed to the idea that they existed as one large panmictic population. However, different lines of evidence indicate the existence of ecological barriers that could have significantly altered sea cow dispersal and population structure, including both periodic (e.g., flooding of Bering Strait during interglacials; glacier advances) and permanent reproductive barriers, such as several wide and deep channels separating the Aleutian Islands.

To evaluate the ecological influence the Steller’s sea cow may have exerted over kelp forests around the North Pacific Rim^11^ we have been preparing updated range maps considering the species glacial and interglacial ranges. These nearshore habitat maps are grounded in the fossil record and guided by bioindicators of kelp habitat, oceanographic data, and various lines of ecological evidence^9–20^ (Fig. 1). Our preliminary range maps indicate that the Commanders population has always been physically isolated from both the population of the Kamchatka peninsula to the West (and the entire Asian mainland population for that matter) and from the nearest source to the East, the Near Islands of the Aleutian chain. Recent reconstructions of Beringia during glacial maxima^12^ confirm that in any scenario of sea level fluctuation the Commanders have always been isolated from the Asian mainland and the Aleutians by deep channels.

**Fig. 1:**
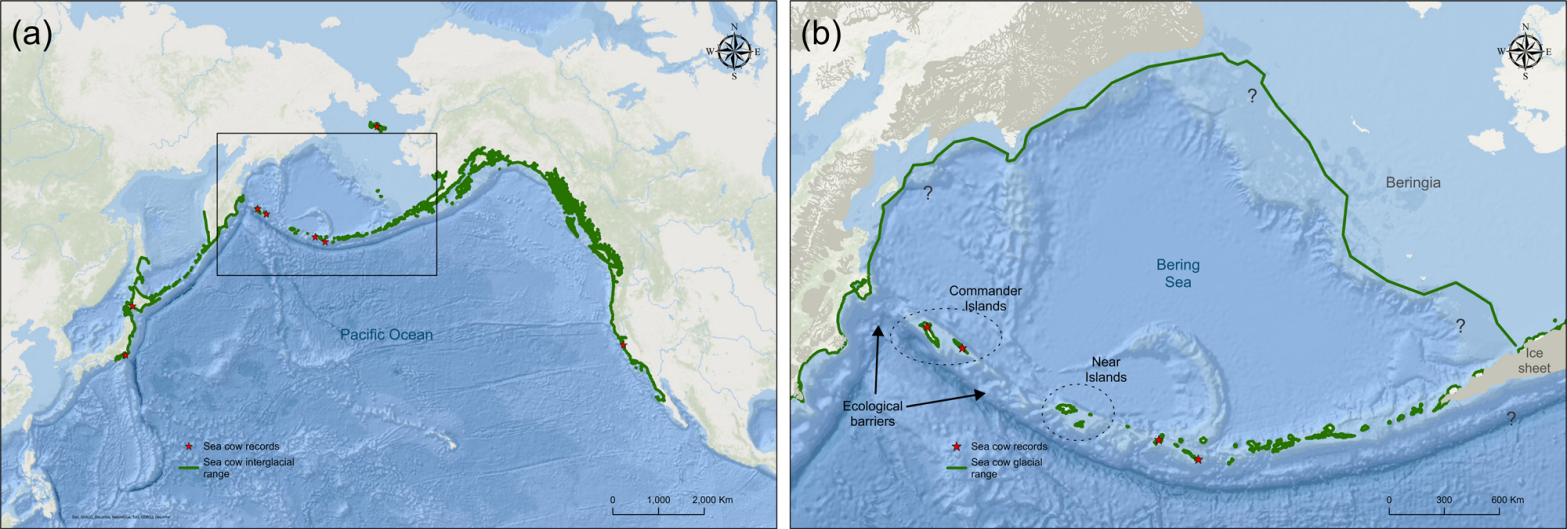
Steller’s sea cow (*Hydrodamalis gigas*) range reconstructions. (**a**) Interglacial range. (**b**) Range during glacial maxima. The Commander Islands have always been physically isolated by deep channels - even during lowest sea levels - that likely constituted ecological barriers, or biogeographic breaks, for sea cows. Different lines of evidence (e.g., absence of echolocation or other navigational apparatus^13^; limited ability to submerge^9^; exclusive seaweed diet^10^) converge to a strictly coastal, ‘linear elongated’ distribution along kelp-dominated shorelines, a range that coincided with that of the sea otter, *Enhydra lutris*^13^. Sea cow range reconstructions were based on: (1) review of existing sea cow records (see Dataset); (2) bioindicators of sea cow habitat (i.e., kelp forests, sea otter historical ranges^14^); (3) southern extent of winter drift ice and year-round kelp habitat;^15,16^ and (4) the estimated shoreline of Beringia and Bering Sea islands during the Last Glacial Maximum^12^. ‘Sea cow records’ (red stars) refer to undisputed records only. Question marks refer to uncertainties related to sea ice extent along the shoreline of Beringia during glacial times. Map background image source and license: Maps were created using ArcGIS Online basemap ‘World Ocean Base’ (Esri, GEBCO, DeLorne, NaturalVue) and using ArcGIS® software by Esri. ArcGIS® and ArcMap™ are the intellectual property of Esri and are used herein under license. Copyright © Esri. All rights reserved. For more information about Esri® software, please visit www.esri.com.

Evidence from sea otter morphological and genetic studies^17^ has shown population structuring across their range and led to the recognition of different subspecies for the Commander Islands and the Aleutian islands^18^ lending support to the idea of ecological barriers at this geographic location. Although we acknowledge considerable differences in life history traits between these species, sea otters are the only other marine mammal restricted to nearshore coastal waters of the North Pacific, sharing a remarkably overlapping distribution with sea cows^19^.

The assessments conducted by Krasheninnikov and Steller in Kamchatka and the Kurile Is., supported by interviews with Indigenous Itelmen and Kurile peoples, did not find ethnobiological evidence of living sea cows outside the Commanders, except for anecdotal reports of carcasses washed ashore^20^. Considering the conspicuousness of the sea cow, this seems to attest to the fact that these animals were hardly, if ever, venturing out of the Commander Is. This is just as we would expect from a strictly coastal sirenian depending on a constant supply of shallow water macroalgae, and possibly freshwater sources^9,10^. Thus, it does not seem that sea cows were either equipped or enticed to cross the 180 km of deep waters and unsuitable habitat between Bering Island and the mainland (or *c*.120 km during glacial maxima) without a shoreline to navigate. The 330 km distance between Medny (the easternmost of the Commander Islands) and Attu (the closest of the Aleutian Islands) would have posed an even greater barrier to genetic exchange. Given the predominant oceanographic conditions, it is possible that an occasional vagrant, or a carcass, as mentioned above, could have floated, or been forced by storms or tsunamis from the Commanders to the Kamchatkan mainland. However, the opposite would have been unlikely given the predominant direction of winds and currents and the low probability of a straggler (floating or swimming) from the continent hitting such a small island on the open ocean.

These assumptions have led us to conclude that the Commander archipelago’s sea cow population—a notoriously relict, stunted population^13^—has likely been genetically impaired from significant contributions from other regions of the Pacific Rim for probably most of its history, possibly for as long as it first colonised the islands, except for the occasional immigrant. In light of these possibilities, we believe that the discussions related to the population history of Steller’s sea cows made by Sharko et al. may be more relevant if related to the context of the Commander Islands population and its peculiar biogeographic trajectory. At the very least, we argue it is not justified to extrapolate results from a single specimen from the Commander Islands population to the rest of the North Pacific given the uncertainty in when they diverged and the degree to which it was genetically isolated. PSMC models may well be able to provide supporting evidence to address these uncertainties, determining when this founding population first established itself in the Commanders, or help elucidate some of the complex dispersal possibilities discussed above.

Another recent paleogenomic study focusing on Steller’s sea cow skin phenotype and cold adaptation has also used PSMC methods to estimate effective population size over time^21^, finding comparable demographic trajectories as Sharko et al. Similarly, since their analyses were only based on individuals from the Commander Islands, their data do not address the question of whether the Commander Islands population accurately represents the demographic history of the species across its former geographic distribution.

Unfortunately, known specimens from outside the Commander Islands are few, and perhaps not able to provide genetic material to allow the level of refinement achieved by Sharko et al. However, the continued development of underwater archaeology and zooarchaeological research may provide future guiding evidence regarding sea cow biogeography and extinction dynamics. In the meantime, similar genetic analyses with existing specimens from California, Alaska (Aleutian and St. Lawrence islands, where other relict populations seem to have persisted through the Holocene) and Japan, may provide a broader perspective on the spatial and demographic dynamics of Steller’s sea cow across the Pacific Rim. In any case, ground-breaking methods such as those used by Sharko et al.—when interpreted in the context of reconstructed species life histories and dispersal dynamics—may constitute one of the best available tools to infer past demographic histories, especially when specimens are rare.

## Reporting summary

Further information on research design is available in the Nature Research Reporting Summary linked to this article.

## Supplementary information

Dataset 1

Reporting Summary

## Data Availability

The data generated in this study, i.e., Steller’s sea cow fossil, sub-fossil and recent records, as well as an annotated list of disputed and undisputed records^22^, have been deposited in the Figshare database under accession code 10.6084/m9.figshare.19891039.v1.
