# Supplementary material for: Steller’s sea cow uncertain history illustrates importance of ecological context when interpreting demographic histories from genomes
Source: Nat Commun. 2022 Jun 28;13:3674. doi: 10.1038/s41467-022-31381-6 (PMC9240004; doi:10.1038/s41467-022-31381-6)
Supplement: Supplementary file 2 — Reporting Summary [file 41467_2022_31381_MOESM2_ESM.pdf]

nature portfolio

Corresponding author(s): Alberto Campos

Last updated by author(s): May 25, 2022

Reporting Summary

Nature Portfolio wishes to improve the reproducibility of the work that we publish. This form provides structure for consistency and transparency in reporting. For further information on Nature Portfolio policies, see our Editorial Policies and the Editorial Policy Checklist.

Statistics

For all statistical analyses, confirm that the following items are present in the figure legend, table legend, main text, or Methods section.

n/a	Confirmed	
☒	☐	The exact sample size (n) for each experimental group/condition, given as a discrete number and unit of measurement
☒	☐	A statement on whether measurements were taken from distinct samples or whether the same sample was measured repeatedly
☒	☐	The statistical test(s) used AND whether they are one- or two-sided<br>*Only common tests should be described solely by name; describe more complex techniques in the Methods section.*
☒	☐	A description of all covariates tested
☒	☐	A description of any assumptions or corrections, such as tests of normality and adjustment for multiple comparisons
☒	☐	A full description of the statistical parameters including central tendency (e.g. means) or other basic estimates (e.g. regression coefficient) AND variation (e.g. standard deviation) or associated estimates of uncertainty (e.g. confidence intervals)
☒	☐	For null hypothesis testing, the test statistic (e.g. F, t, r) with confidence intervals, effect sizes, degrees of freedom and P value noted<br>*Give P values as exact values whenever suitable.*
☒	☐	For Bayesian analysis, information on the choice of priors and Markov chain Monte Carlo settings
☒	☐	For hierarchical and complex designs, identification of the appropriate level for tests and full reporting of outcomes
☒	☐	Estimates of effect sizes (e.g. Cohen's d, Pearson's r), indicating how they were calculated

Our web collection on statistics for biologists contains articles on many of the points above.

Software and code

Policy information about availability of computer code

Data collection	No software was used for data collection.
Data analysis	The commercially-available GIS (Geographic Information Systems) software ArcGIS Pro version 10.8.2 (by ESRI) was used to prepare preliminary layout maps (license number for Alberto Campos #1888011411).

For manuscripts utilizing custom algorithms or software that are central to the research but not yet described in published literature, software must be made available to editors and reviewers. We strongly encourage code deposition in a community repository (e.g. GitHub). See the Nature Portfolio guidelines for submitting code & software for further information.

Data

Policy information about availability of data

All manuscripts must include a data availability statement. This statement should provide the following information, where applicable:

- Accession codes, unique identifiers, or web links for publicly available datasets
- A description of any restrictions on data availability
- For clinical datasets or third party data, please ensure that the statement adheres to our policy

An original dataset with the published fossil and sub-fossil records for Steller's sea cow has been compiled for this research, including their collectors, museum collection, collection code/number, type of specimen/tissue, locality, geographic coordinates, age of specimen and reference(s). This dataset is publicly available as described in the Supplementary Information/Source Data section.

March 2021

Field-specific reporting

Please select the one below that is the best fit for your research. If you are not sure, read the appropriate sections before making your selection.

☐ Life sciences ☐ Behavioural & social sciences ☒ Ecological, evolutionary & environmental sciences

For a reference copy of the document with all sections, see nature.com/documents/nr-reporting-summary-flat.pdf

Ecological, evolutionary & environmental sciences study design

All studies must disclose on these points even when the disclosure is negative.

Study description	This study is part of an ongoing effort to evaluate the ecological roles that Steller's sea cows may have exerted over kelp forest ecosystems of the North Pacific Rim (see Bullen et al. 2021; https://onlinelibrary.wiley.com/doi/10.1111/geb.13370) and their social-ecological implications. Based on the fossil record, Steller's descriptions and accounts, and the extent of kelp habitat, we are estimating sea cow glacial and interglacial ranges, population size and density, in order to evaluate the consequences of their absence, and discuss possibilities to restore some of these ecological interactions.
Research sample	To estimate glacial and interglacial ranges of the Steller's sea cow, and be able to build an accurate range map for the species in order to properly support our assumptions, we have compiled a dataset with all the existing fossil and sub-fossil records for the Steller's sea cow across its North Pacific distribution. This research sample represents 100% of the published fossil and sub-fossil records for the Steller's sea cow. This dataset is publicly available as described in the Supplementary Information/Source Data section.
Sampling strategy	No sample calculation was performed. All published records were computed and expected to encompass 100% of the samples available.
Data collection	To collect the data, a comprehensive literature search was conducted by the corresponding author [AC]. The data was organized and stored in a spreadsheet as a database, including: locality, type of evidence, collector, museum collection number, sample age.
Timing and spatial scale	Data was collected from August 2019 to August 2021. Geographically, data were taken from the North Pacific Rim, from Japan to California (US), including the Aleutian chain of islands. The samples covered roughly the period of the Late Pleistocene and Holocene Epoch, i.e., from 130,000 years ago to present.
Data exclusions	Six data points were excluded from the analyses, because they are presently disputed: (1) two samples from the Alaskan mainland because they were re-worked and found in a cultural context; and (2) four samples from the Aleutian islands, because their identification was later questioned, or proved to be wrong by DNA and/or isotope bone analyses.
Reproducibility	There were no experiment to test for reproducibility.
Randomization	This does not seem relevant, since we have expected to include 100% of the existing samples.
Blinding	Blinding does not seem relevant in this case, since we included all the samples available and there was little subjectivity during data analyses.

Did the study involve field work? ☐ Yes ☒ No

Reporting for specific materials, systems and methods

We require information from authors about some types of materials, experimental systems and methods used in many studies. Here, indicate whether each material, system or method listed is relevant to your study. If you are not sure if a list item applies to your research, read the appropriate section before selecting a response.

Materials & experimental systems

n/a	Involved in the study	
☒	☐	Antibodies
☒	☐	Eukaryotic cell lines
☒	☐	Palaeontology and archaeology
☒	☐	Animals and other organisms
☒	☐	Human research participants
☒	☐	Clinical data
☒	☐	Dual use research of concern

Methods

n/a	Involved in the study	
☒	☐	ChIP-seq
☒	☐	Flow cytometry
☒	☐	MRI-based neuroimaging
